# The jam-based discovery framework: How lab culture, shared data and collaboration shape scientific discovery

**DOI:** 10.1038/s44319-025-00686-0

**Published:** 2026-01-03

**Authors:** Roy Maimon

**Affiliations:** 1 Department of Biomedical Engineering, New York University, Tandon School of Engineering, New York City, NY USA; 2 Department of Cellular and Molecular Medicine, University of California at San Diego, La Jolla, CA USA

**Keywords:** History & Philosophy of Science, Methods & Resources

## Abstract

Science and music are improvisational acts built on structure, discipline and intuition. And like music, science advances when ideas flow freely, when mistakes become motifs and when everyone in the room listens.

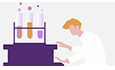

## Main

Queen’s and David Bowie’s *Under pressure*, Jimi Hendrix’s *Voodoo Child* and even Ed Sheeran’s *Shape of You* began in jam sessions: moments when artists from different backgrounds played freely until something new emerged. A jam session is not chaos though. While it is freedom in rhythm and structure and relies on spontaneity (Bengtsson et al, 2007; Limb and Braun, 2008), all players pay attention to each other, react to the flow of music and create something new.

### The logic of jam-based discovery

A scientific jam session is not much different. Musicians have drums and guitars; scientists have pipettes, sequencers, and microscopes. Their figures and datasets are compositions shaped by practice, intuition, and collaboration. The scientific paper is the official studio or concert recording: the polished, final performance. But the real breakthroughs happen in rehearsal, when the unexpected is allowed to surface. Yet, academic science often treats cooperation and creativity as an afterthought, something that happens in spite of structure, not because of it, even though models of collective creativity in science argue that lab culture and structure are precisely what enable breakthrough ideas (Neumann, 2007). Creativity and inspiration emerge when researchers from different disciplines gather for an open exchange of data, ideas and prototypes—a scientific jam session.

“The real breakthroughs happen in rehearsal, when the unexpected is allowed to surface.”

In this essay, I use “jam-based discovery framework” to describe a simple pattern: scientists meet in short cycles to discuss and analyse their data and experiments, and record every step so that ideas and failures remain accessible for future use. The inputs are shared space, heterogeneous expertise and data. The process is disciplined improvisation under agreed constraints. The outputs are small but cumulative: new analyses, improved experiments and controls, and reframed hypotheses that can be tested and refined.

There is a difference to musical jam sessions, though. Musicians improvise to create sound and are free to play whatever they are up to. Scientists improvise to solve a problem, explain an observation or test a hypothesis. A scientific jam is therefore not freeform play; it is structured improvisation towards a commonly shared goal. The creativity resembles a jam, but the purpose remains firmly problem-oriented: to generate actionable next steps such as a new analysis, a sharper hypothesis or an experimental design that helps to clarify the question. It is what occurs when, for instance, a computational biologist, an engineer and a neuroscientist sit around, open their laptops and pursue an idea.

As highlighted in the book *Outsider Scientists: Routes to Innovation in Biology*, randomness and foreign perspectives are needed for discovery. They help to find the misplaced note or raw idea that can make the difference. Modern science often discourages this randomness in favor of controlled precision. Both are needed, however. Improvisation without discipline is chaos; discipline without improvisation is stagnation. Improvisation lowers the cognitive load associated with perfectionism, enabling more diverse hypothesis generation (Felsman et al, 2020); improvised-based interventions have been used to support creativity, engagement and psychological well-being in older adults (Keisari et al, 2024).

“Improvisation without discipline is chaos; discipline without improvisation is stagnation.”

Through my PhD, I came close to becoming a professional drummer. I stayed in science, but the studio routine never went away. Both fields need discipline, rehearsal and taste. Both require listening, timing and knowing when to lead or when to hold back. Importantly, both depend on spaces that encourage collaboration and capture inspiration before it gets away. The best labs don’t just test hypotheses; they construct tools, methods and environments where new ideas can take shape.

### How scientific jam sessions emerge

Just as every musician brings their personal timing, every scientist has a biological and cognitive rhythm. Environments that respect these individual “bio rhythms” tend to generate more original ideas, while rigid or abusive cultures quickly silence creative risk-taking. A true scientific jam session, therefore, requires not only technical structure but also psychological safety, so that students and postdocs can try new directions without fear of humiliation or retaliation.

“Environments that respect these individual “bio rhythms” tend to generate more original ideas, while rigid or abusive cultures quickly silence creative risk-taking.”

The jam in science moves the creative part from individuals to collectives. It requires scientists who share a common language yet differ enough to bring different perspectives and ideas to the table (Uzzi et al, 2013). A synthetic biologist and a data scientist can jam, a physicist and an engineer can improvise if their equipment can communicate. Artificial intelligence adds a new collaborator today, just like Auto-Tune did in the late 1990s: an algorithmic “bandmate” that can recognize motifs within data faster than people can see them and suggest new directions.

For example, in our early interdisciplinary meetings between New York University (NYU) Tandon engineers and NYU Langone neuroscientists, the “jam session” often begins with a single unresolved dataset projected on a shared screen. An imaging scientist might annotate a puzzling structural feature, a computational biologist might write a quick script to quantify it, and a clinician may explain how such a pattern appears in patients. Within an hour, a rough analysis emerges that none of the groups could have produced independently. No grant was written, no manuscript planned, yet a new direction becomes visible simply because people were allowed to improvise around real data in real time. This is what the scientific jam looks like when it happens inside and between labs.

### Design principles for scientific jam sessions

A jam-based discovery framework does not create itself even if people have good intentions and interest. It requires support and design to make it happen. Here are five simple principles that help to encourage open discussion and improvisation.

#### Architecture

Remove physical and digital barriers. Open space allows interdisciplinary cross-talk (Bernstein and Turban, 2018). A biologist listening in on a computation discussion may hear a pattern nobody coded in. Shared kitchens, common writable walls, mixed seating plans, and open-data dashboards encourage unplanned encounters and side conversations as part of the design rather than as an accident. Science advances most when people from different “instruments” such as neuroscience, physics, computer science, and engineering share a space where it is safe to play wrong notes.

#### Record every take

Capture the process, not just the outcomes. Studios record every rehearsal. Labs often erase their creative iterations and retain only the final results. In a jam-based lab, all scientific jams are recorded, versioned and archived: whiteboard snapshots, exploratory notebooks, pilot scripts, and “failed” analyses. This makes reproducibility a built-in feature rather than something done after the fact. It is not about surveillance. It is about making creative thinking retrievable when a new idea builds on the last.

#### Culture of improvisation

Normalize visible thinking. Transparency about the process adds credibility to the enterprise and encourages creativity. An open jam permits replay of the tune, learning from the riff and developing the melody into new material. Models of collective creativity in science emphasize that cultures of openness, mutual respect, and shared authorship are exactly what allow novel ideas to emerge. Team-science studies show that collaborations combining diverse expertise consistently yield higher impact work than homogeneous teams or solo investigators (Wuchty et al, 2007).

#### Rhythm

Run rapid, low-cost cycles. Jams move in short phrases: play, listen, adjust. In the lab, this translates into rapid cycles of hypothesis, experiments and immediate feedback. Teams hold brief “idea jams” where one slide or one experiment is explored for 20 min, then turned into a concrete next step: a quick script, a small pilot or a new control. These short cycles lower the stakes of being wrong and keep the tempo of discovery high.

#### Harmony

Rotate who leads and who supports. Bands sound at their best when leadership can move between players. The same is true in labs. Passing the lead means giving early-career researchers space to propose and drive ideas, not just to execute them. It means publishing negative results that close loops (Nimpf and Keays, 2020), and recognizing the session players, technicians, analysts and engineers whose skills make the track sound good. Leadership here is structured awareness and respect, not loudness: integrating diverse expertise in the same room and allowing the “solo” to move as problems change.

### Where discovery takes shape

**Figure 1:**
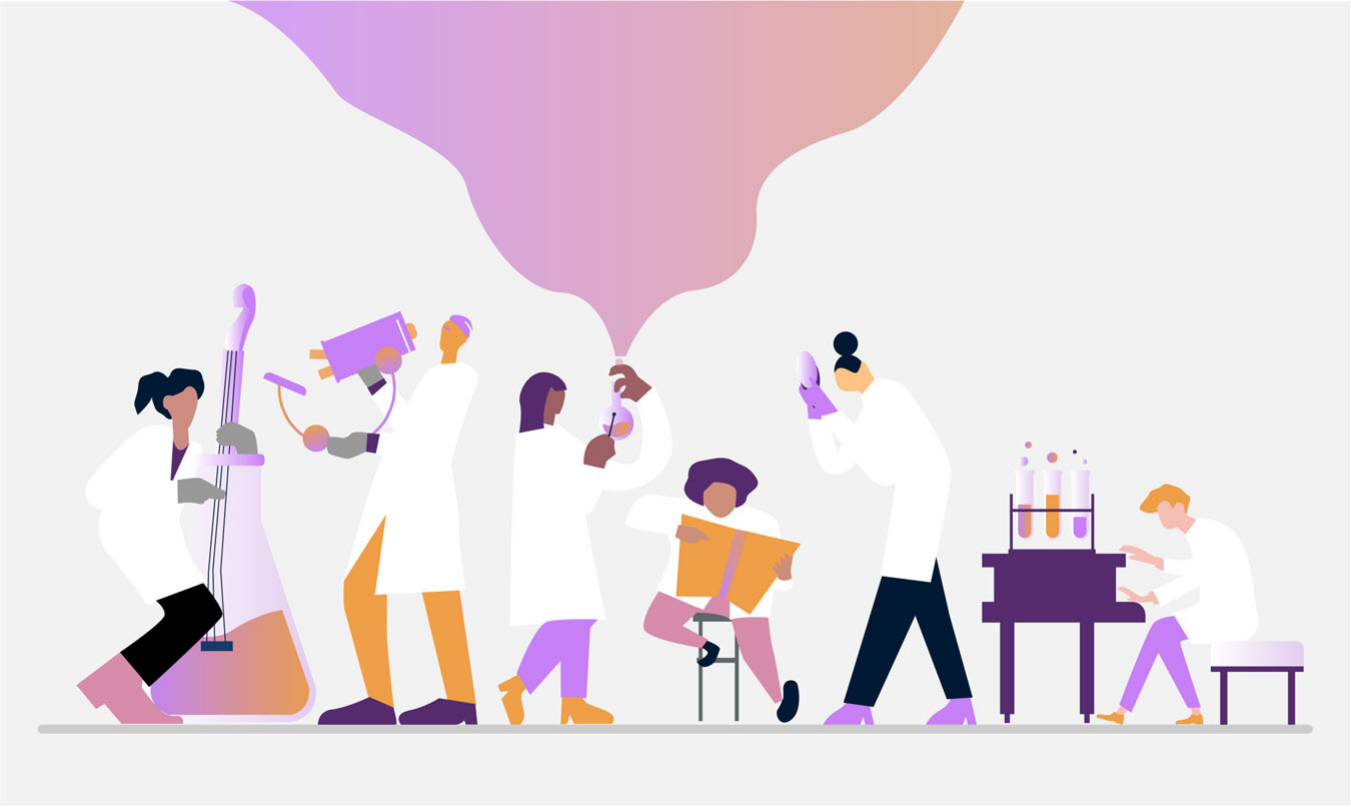
When Science Jams. Rather than following rigid experimental scripts, scientific jam sessions emphasize improvisation, dialogue, and shared exploration.

Science’s most revolutionary breakthroughs have always begun as jams. Pasteur’s swan-neck flask demonstrated that life does not emerge spontaneously but requires nutrients to thrive (Pasteur, 1861). Hodgkin and Huxley’s voltage-clamp amplifier gave the squid axon a voice (Hodgkin and Huxley, 1952). In my previous lab, some of the best ideas emerged exactly during a jam. At one point, our imaging pipeline stalled because the custom glass slides were too large and too thin for the microscope stage. Instead of ordering new and expensive hardware, we sat around the bench and improvised a solution, eventually deciding to bring in a diamond cutter and reshape the slides ourselves. In another case, spatial transcriptomics experiments were bogged down by hours of repetitive pipetting, so the biologists, engineers and programmers in the group met at the whiteboard and designed a small, automated pipetting device that took over the most tedious steps. Neither solution came from a formal meeting nor a single person. They appeared in real time when different experts discussed a shared problem (Fig. 1).

To facilitate this ethos, once a luxury, we need funding models that value and support it. Grants should include a small, straightforward percentage for exploratory research based on structured improvisation with clear requirements for documentation and open data. Reviewers should not only judge outcomes but also a lab’s capacity to create new, open collaboration. Journals need to reward co-shared process notes, code and protocols along with figures. The labs that master improvisational cycles will outpace traditional labs in discovery speed.

“The labs that master improvisational cycles will outpace traditional labs in discovery speed.”

Science, like music, is a social art that is both disciplined and improvisational. The jam session is where new motifs are born, where newcomers can change the beat, and where passion bumps up against precision. For laboratories, this translates into concrete design choices: shared spaces that enable encounters, data systems that record exploratory work, fair credit for all contributors, and a culture that rules out abusive behavior and treats everyone the same. Creativity becomes sustainable only when these conditions are treated as infrastructure, not as afterthoughts. To embrace it, one can plan laboratories as studios, but the more fundamental goal is cultural: to make creativity a habitual practice, not a caprice. Our task as scientists is clear: chart the diagram, share the track and challenge others to play the subsequent chorus. Science is not only a protocol, it is a performance. We need to redesign the studio.

“Creativity becomes sustainable only when these conditions are treated as infrastructure, not as afterthoughts.”

“Science is not only a protocol, it is a performance.”

## Supplementary information

Peer Review File

## References

[CR1] Bengtsson SL, Csikszentmihalyi M, Ullen F (2007) Cortical regions involved in the generation of musical structures during improvisation in pianists. J Cogn Neurosci 19:830–842 17488207 10.1162/jocn.2007.19.5.830

[CR2] Bernstein ES, Turban S (2018) The impact of the ‘open’ workspace on human collaboration. Philos Trans R Soc Lond B Biol Sci 373:20170239 29967303 10.1098/rstb.2017.0239 PMC6030579

[CR3] Felsman P, Gunawardena S, Seifert CM (2020) Improv experience promotes divergent thinking, uncertainty tolerance, and affective well-being. Think Ski Creat 35:100632

[CR4] Hodgkin AL, Huxley AF (1952) A quantitative description of membrane current and its application to conduction and excitation in nerve. J Physiol 117:500–544 12991237 10.1113/jphysiol.1952.sp004764 PMC1392413

[CR5] Keisari S, Krueger KR, Ben-David BM, Hainselin M (2024) New horizon in improving ageing with improvisational theatre. Age Ageing 53:afae087 38706392 10.1093/ageing/afae087

[CR6] Limb CJ, Braun AR (2008) Neural substrates of spontaneous musical performance: an FMRI study of jazz improvisation. PLoS ONE 3:e1679 18301756 10.1371/journal.pone.0001679 PMC2244806

[CR7] Neumann CJ (2007) Fostering creativity. A model for developing a culture of collective creativity in science. EMBO Rep 8:202–206 17330061 10.1038/sj.embor.7400913 PMC1808036

[CR8] Nimpf S, Keays DA (2020) Why (and how) we should publish negative data. EMBO Rep 21:e49775 31858691 10.15252/embr.201949775 PMC6945059

[CR9] Pasteur L (1861) Mémoire sur les corpuscules organisés qui existent dans l’atmosphère, examen de la doctrine des générations spontanées. Ann Sci Nat Zool 16:598

[CR10] Uzzi B, Mukherjee S, Stringer M, Jones B (2013) Atypical combinations and scientific impact. Science 342:468–472 24159044 10.1126/science.1240474

[CR11] Wuchty S, Jones BF, Uzzi B (2007) The increasing dominance of teams in production of knowledge. Science 316:1036–1039 17431139 10.1126/science.1136099

