# Supplementary material for: The jam-based discovery framework: How lab culture, shared data and collaboration shape scientific discovery
Source: EMBO Rep. 2026 Jan 3;27(3):570–2. doi: 10.1038/s44319-025-00686-0 (PMC12895035; doi:10.1038/s44319-025-00686-0)
Supplement: Supplementary file 1 — Peer Review File [file 44319_2025_686_MOESM1_ESM.pdf]

EMBO reports

# The Jam-Based Discovery Framework

Roy Maimon

*Corresponding author(s): Roy Maimon (roy.maimon@nyu.edu)*

## Review Timeline:

| | |
|---|---|
| Submission Date: | 10th Dec 25 |
| Accepted: | 18th Dec 25 |

*Editor: Holger Breithaupt*

## Transaction Report:

This article was editorially reviewed.
